# Enhanced Cognitive Behavioral Therapy for Young Adolescents with Anorexia Nervosa: Identifying Predictors of Treatment Response

**DOI:** 10.3390/nu17172731

**Published:** 2025-08-23

**Authors:** Simona Calugi, Mirko Chimini, Anna Dalle Grave, Gianmatteo Cattaneo, Maddalena Conti, Riccardo Dalle Grave

**Affiliations:** Department of Eating and Weight Disorders, Villa Garda Hospital, Via Monte Baldo, 89, 37016 Garda, VR, Italy; si.calugi@gmail.com (S.C.); mirko.chimini1@gmail.com (M.C.); annadallegrave@gmail.com (A.D.G.); gianmatteocattaneo@gmail.com (G.C.); missmadda@hotmail.com (M.C.)

**Keywords:** anorexia nervosa, adolescents, cognitive behavior therapy, predictors, full response, inpatient treatment, cohort study, prospective study

## Abstract

(1) Background: This study aimed to identify baseline demographic, clinical, and psychosocial predictors of treatment response in adolescents with anorexia nervosa (AN) undergoing an intensive 20-week enhanced cognitive behavioral therapy (CBT-E) program, which included inpatient and day patient phases. Treatment outcomes were assessed at the end of intensive treatment (EOIT) and at a 20-week follow-up. (2) Methods: A prospective cohort of 68 adolescents under the age of 16 consecutively admitted to intensive CBT-E was evaluated. Baseline measures included body mass index (BMI)-for-age percentiles, percentage of expected body weight (%EBW), eating disorder psychopathology (EDE-Q), general psychopathology, and functional impairment. (3) Results: Of those who began treatment, 83.4% completed the program and 70.2% were available for follow-up assessment. Based on intent-to-treat analysis, 94.1% achieved a “good BMI outcome” and 73.5% met criteria for “full response” at EOIT. At follow-up, 64.7% maintained a good BMI and 55.9% sustained a full response. Completers’ analysis indicated that baseline body weight, clinical impairment, general psychopathology, and weight regain influenced treatment outcomes. However, no baseline demographic or clinical variables predicted treatment completion or outcome at either time point at intention-to-treat analysis, except that younger age at admission was linked to higher eating disorder psychopathology at follow-up. (4) Conclusions: In treatment completers, certain baseline clinical factors and weight regain influenced outcomes, while in the full sample, younger age predicted greater residual psychopathology at follow-up. These findings, if confirmed, emphasize the need for early intervention, focused support for weight regain, and potential adaptations of CBT-E for early adolescents.

## 1. Introduction

Anorexia nervosa (AN) typically emerges during adolescence and can be severe impairment in physical functioning, significant medical complications, reduced quality of life, and an elevated risk of mortality [1,2]. Retrospective analyses suggested that a longer duration of untreated AN strongly predicts poorer outcomes [3], These results underscore the critical need to develop tailored interventions specifically for adolescents with AN, as well as the importance of identifying key predictors of treatment response in this population.

Nevertheless, research investigating factors that predict treatment outcomes in adolescents with AN has produced inconsistent results. The present literature found associations between poorer outcomes and factors such as low body weight at admission, heightened symptom severity [4,5,6,7,8,9,10,11,12], younger age [9,13] or older age [14], co-occurring psychiatric conditions [9,12], elevated parental expressed emotion [15], low perceived parental control [16], and early weight gain or other symptom improvement or weak therapeutic alliance [4,7,11,17,18,19,20].

In contrast, other investigations have reported no significant associations between outcomes and variables such as baseline weight, symptom severity, family dynamics, or comorbid psychopathology [5,8,21,22]. Findings related to depression and anxiety have also been inconclusive: while some research indicates that lower baseline levels predict more favorable outcomes [12,23], other have shown that greater initial levels may correspond to better results in certain patient subgroups [12,13,24].

Notably, these studies primarily focus on usual care in inpatient or day-patient settings, or on family-based treatment (FBT). In contrast, research investigating predictors of treatment response in adolescents receiving enhanced cognitive behavioral therapy for anorexia nervosa (CBT-E) remains scarce. CBT-E is a treatment recommended by the National institute for Health and Care Excellence when FBT is unacceptable, contraindicated or ineffective [25]. To date, only one study has explored predictors of outpatient CBT-E outcomes in adolescent outpatients [26], reporting no baseline variables as significant predictors of treatment response or dropout. Similarly, the sole investigation of predictors of outcome in inpatient CBT-E for adolescents considered only a single factor—duration of illness—and found no association between illness duration (≤ or >3 years) and either body weight or eating disorder psychopathology at end of treatment or follow-up [27]. Finally, a recent study found significant predictors in a group of patients treated in intensive CBT-E with the diagnosis of anorexia nervosa, but including only patients over 16 years [28].

Given this significant gap, the main aim of the present study is to evaluate predictors of treatment outcomes in adolescents aged 16 years or younger with AN who received intensive CBT-E.

## 2. Materials and Methods

### 2.1. Design

This prospective cohort study enrolled adolescents under the age of 16 years who were consecutively admitted to the Department of Eating and Weight Disorders at Villa Garda Hospital (Italy) between January 2016 and March 2024. The study has been ethical approved from the GHC Institutional Review Board (Protocol No. 0022GHCIRB, 10 February 2025). All study participants and their parent(s) or legal guardian(s) signed a written informed consent, authorizing the collection and anonymized use of clinical data for research purposes.

### 2.2. Participants

All patients were referred to the unit by general practitioners or secondary care providers. All were first-time admissions to the Villa Garda Eating Disorder Unit, except for two individuals who had previously been treated at the same facility. Half of the sample (50%) had prior hospitalizations for medical stabilization in general pediatric or medical wards, and 56.3% had received treatment in other inpatient eating disorder facilities.

Eligibility criteria for admission included: (i) age between 13 and 65 years; (ii) fulfilling diagnostic criteria for an eating disorder as defined by DSM (American Psychiatric Association, 2013); (iii) inability to manage the disorder safely in an outpatient setting; and (iv) at least one unsuccessful attempt at outpatient treatment. Exclusion criteria were: (i) current substance abuse; and (ii) active psychotic or bipolar disorders.

Between January 2016 and March 2024, 655 patients were hospitalized in the Unit. Of these, 68 adolescents (10.4%) met the inclusion criteria of being under 16 years old with a DSM-5 diagnosis of anorexia nervosa. Among them, 57 patients (83.8%) completed the full treatment program, while 11 (16.2%) discontinued prematurely. Treatment drop-out was defined as ending therapy before completing the scheduled course, due to factors such as low motivation, dissatisfaction with treatment, perceived lack of progress, or ambivalence toward recovery.

At the 20-week post-treatment follow-up, 40 of the 57 treatment completers (70.2%) participated in the follow-up assessment.

### 2.3. The Treatment

The intervention implemented in this study was based on CBT-E, specifically adapted for adolescent patients in accordance with evidence-based protocols developed at Villa Garda [29,30].

The program lasts 20 weeks, divided into 13 weeks of inpatient phase and followed by 7 weeks of day-hospital treatment. Both components were delivered at Villa Garda Hospital, located in a small town in northern Italy, a publicly funded facility linked to the National Health Service.

Admission was voluntary and preceded by four preparatory sessions led by a psychologist trained in CBT-E. These sessions aimed to present the treatment model, distinguish between medical and CBT-E psychological approaches, build motivation for recovery, and secure the patient’s commitment to begin weight restoration from the beginning of hospitalization.

The intervention was delivered in an open-unit setting, enabling patients to confront real-life environmental challenges with the support of a specialized, non-eclectic multidisciplinary team. This team—comprising psychologists, physicians, nurses, dietitians, and physical therapists—was uniformly trained and supervised in CBT-E [29] and participated in weekly case-management meetings to ensure individualized, coherent care.

CBT-E was delivered through a combination of individual and group sessions. Individual sessions were conducted twice a week for the first four weeks and weekly for the rest of the treatment. Group CBT-E sessions occurred four times per week. A CBT-E-trained dietitian supported patients during meals in the early treatment phase, while medical and nursing staff monitored physical health, conducted collaborative weigh-ins, and provided crisis support.

Following the transdiagnostic model, the treatment targeted both the focus of psychopathology of eating disorders—namely, the overvaluation of shape, weight, and eating control—and maintaining mechanisms such as low body weight, dietary restriction and restraint, events, and associated mood changes conditioning eating, using structured CBT-E modules.

The central focus of the initial phase of CBT-E (Step One) is to work on engagement, motivation-building, and helping patients understand their eating disorder using a personalized cognitive-behavioral formulation. Addressing weight regain, dietary restriction, and other extreme weight control behaviors, in-session weighing, and psychoeducation are also introduced in this early phase. In the middle phase (Step Two), treatment addresses the key maintaining mechanisms of the disorder—such as overvaluation of body shape and body weight, dietary restraint, events and associated mood changes conditioning eating. In the last phase (Step Three), the aim is to consolidate the changes achieved and minimize the risk of relapse after discharge. This plan includes strategies to respond effectively to potential setbacks, maintain new habits, and recognize early warning signs of relapse. In addition, the team dietitian assists with meal planning at home.

With adolescent patients, parents and/or legal guardians are also involved in joint and separate sessions, as indicated by the CBT-E for adolescents, to enhance family support, to promote a positive home environment, to facilitate adherence to treatment goals, and to assist with relapse prevention planning, particularly toward the end of treatment. This is an opportunity for the family members to improve communication between each other and to co-develop functional strategies for handling dysfunctional eating behaviors and crises.

At the end of the intensive program, patients are offered 20 optional outpatient CBT-E sessions. These sessions are designed to consolidate progress, address residual psychopathology, and reinforce relapse-prevention skills and are supplied in a period of 20 weeks.

Overall, intensive CBT-E offers a structured, individualized, and evidence-based intervention for adolescents with anorexia nervosa who have not responded adequately to outpatient treatment. It emphasizes active patient engagement, a collaborative therapeutic relationship, and constructive family environment, to achieve a complete and sustained recovery.

### 2.4. Assessment

Validated, standardized instruments were used to assess treatment outcomes at admission (baseline), end of intensive treatment (EOIT), and 20-week follow-up (20-week FU).

- Demographic and clinical characteristics were collected via interview, including age of onset. Body weight and height were surveyed using calibrated equipment (floor scale and wall-mounted stadiometer) on the first day of admission, at discharge, and at follow-up with patients wearing only underwear. BMI-for-age percentiles were calculated using CDC growth charts [31] (https://www.cdc.gov/growthcharts/cdc-growth-charts.htm, accessed on 10 January 2025) BMI-for-age percentiles below one were recorded as 0.5. Expected body weight (EBW) was computed employing the BMI method [32].
- Eating disorder psychopathology was evaluated using the Italian version of the Eating Disorder Examination Questionnaire (EDE-Q, 6th edition) [33,34]. The internal consistency in the study sample was 0.92.
- General psychopathology was measured using the Brief Symptom Inventory (BSI) in the Italian adaptation [35,36], which the Cronbach alpha in our sample was 0.97. Although the BSI is primarily used in adult populations, independent researchers were present during the administration of all questionnaires to clarify any uncertainties about item meanings for younger participants.
- Functional impairment related to eating disorder symptoms was evaluated using the Italian adaptation of the Clinical Impairment Assessment (CIA) [37,38]. The internal consistency of the instrument was also strong (Cronbach’s α = 0.92).

### 2.5. Outcome Categories

In the current study were adopted two operational outcome categories:

1. “Good BMI outcome”: Achieving a BMI-for-age percentile equivalent to an adult BMI of ≥18.5 kg/m^2^, representing the lower threshold of a healthy BMI range [39,40].
2. “Full response”: Meeting both the Good BMI Outcome criteria and having a global EDE-Q score below 2.77, which is less than one standard deviation above the community mean [41].

### 2.6. Statistical Analysis

To assess changes in clinical outcomes over time, mixed-effects models were used for each primary outcome measure: percentage of EBW, BMI-for-age percentile, EDE-Q global and subscale scores, BSI global score, and CIA global score. Time was modeled as a fixed effect, while participant-level random effects accounted for individual variability, assuming participants were a random sample from a broader treatment-seeking adolescent AN population. A two-level hierarchical structure was specified, with repeated measurements nested within individuals. Maximum likelihood estimation was applied [42]. To capture potential nonlinear trajectories, quadratic time trends were tested [43,44].

To address missing data on BMI-for-age percentile, EDE-Q (both global and subscale scores), BSI global score, and CIA global score at both the end of intensive treatment (EOIT) and 20-week follow-up (FU), a multiple imputation approach was employed using fully conditional specification (FCS). This process, appropriate for arbitrary patterns of missingness, was used in combination with Predictive Mean Matching to preserve the distributional properties of the data [45]. The proportion of missing data was 16% at EOIT and 41.1% at follow-up.

Multivariable logistic regression models were employed to identify predictors of treatment drop-out, good BMI outcome, and full response at EOIT and after 20-week follow-up. Predictor variables included baseline characteristics (age, duration of illness, baseline BMI-for-age percentile, EDE-Q, BSI, and CIA global scores) as well as changes from baseline to EOIT in EDE-Q, BMI, BSI, and CIA scores.

In addition, linear regression analyses were conducted to explore which baseline or change variables predicted BMI-for-age percentile and eating disorder psychopathology (EDE-Q global score) at EOIT and follow-up.

Regression analyses were run both in patients who completed 20-week FU (per-protocol analysis) and as an intention-to-treat analysis.

In order to evaluate the missing-at-random (MAR) assumption, a multivariable logistic regression was carried out using “missingness” (yes/no) at EOIT as the dependent variable. Baseline predictors included duration of illness, age, BMI-for-age percentile, and global scores on the EDE-Q, BSI, and CIA. No significant associations were found, suggesting that missingness was unrelated to observed baseline characteristics. Similarly, no significant differences were found among participants who completed the follow-up phase and those participants who did not (all *p*-values > 0.05). Based on this, five imputed datasets were generated, and pooled estimates were used for all subsequent analyses.

Descriptive statistics are presented as means and standard deviations (SD) or standard error (SE) or as frequencies and percentages. All analyses were conducted using IBM SPSS Statistics, Version 29.0 (Released 2024; IBM Corp., Armonk, NY, USA).

## 3. Results

### 3.1. The Sample

The sample included 68 female adolescent patients with AN. Main demographic and clinical characteristics of the sample are shown in Table 1: mean age = 14.6 years (SD = 1.0, range 11–15.99 years), mean BMI-for-age percentile = 5.0 (SD = 7.6; range 0.4–40.5), and mean duration of illness = 1.8 years (SD = 1.3; range: 0–8 years).

### 3.2. Intent-to-Treat Findings at EOIT and 20-Week FU

Fifty-seven (83.8%) finished the intensive CBT-E program. Of those, 40 (70.2%) participated in follow-up interviews at 20 weeks. About 97.5% of follow-up responders received a post-discharge treatment, with 89.7% of those patients receiving a 20-week CBT-E-based treatment. The latter was carried out by trained therapists living close to their place of residence.

Linear mixed models indicated an initial significant increase in BMI-for-age percentile and EBW (linear growth) during the treatment, and a subsequent deceleration in the rate of change (quadratic growth) during the follow-up. Both EDE-Q subscale and global scores and CIA global scores replicated this pattern, all significantly decreasing during the treatment and then showing a deceleration in the rate of change at 20-week FU. BSI global score showed a significant linear improvement from baseline to 20-week FU (Table 2).

Among the 68 patients assessed at baseline, 94.1% achieved a good BMI outcome, while 73.5% experienced a full response at EOIT. At the 20-week FU, 64.7% kept a “good BMI outcome” and 55.9% remained in “full response”.

Considering 40 patients who completed the 20-week FU assessment, 39 (97.5%) reached a “good BMI outcome” and 31 (77.5%) a “full response” at EOIT. These cut-offs were reached by 67.5% and 55%, respectively, at the 20 weeks of follow-up.

### 3.3. Predictors of Treatment Outcome (Per-Protocol and Intention-to-Treat Analysis)

Per-protocol analysis included 40 adolescent patients who completed the 20-week FU assessment. Completer patients who reached “good BMI outcome” and “full response” at EOIT are 39 out of 40 (97.5%) and 31 out of 40 (77.5%), respectively. Considering the very small number of patients who did not reach these cut-offs, we have decided to show logistic regression analysis only for dichotomous treatment outcomes at follow-up. On the contrary, linear regression analysis was performed for continuous outcomes both at EOIT and 20-week FU (see Table 3a,b).

Completers’ logistic analysis indicated that each additional BMI-for-age percentile point increases the likelihood of achieving a “good BMI outcome” by more than threefold, at follow-up. No other predictors of “good BMI outcome” and of “full response” were found.

Linear regression indicated that the baseline CIA predicted both BMI-for-age percentile and EDE-Q at EOIT. In particular, lower clinical impairment perceived at baseline predicted higher BMI-for-age percentile and lower eating disorder psychopathology at the end of treatment. However, this finding is not confirmed at follow-up, where higher BMI-for-age was predicted by higher baseline general psychopathology and larger change in BMI-for-age percentile from baseline to EOIT, and lower EDE-Q global score was predicted by lower BSI global score measured at baseline.

Intention-to-treat analysis did not confirm the per-protocol analysis showing that none of the demographic or baseline clinical variables were significantly associated with the drop-out rate, with “good BMI outcome”, with “full response” or with BMI-for-age percentile and EDE-Q global score both at EOIT and at 20-week FU. The only exception was the baseline age, which was a significant predictor at 20-week FU of eating-disorder psychopathology (beta = −0.38, *t*-test = 2.07, *p* = 0.039, 95% CI: −0.75–−0.02). A lower age predicted higher EDE-Q global score at follow-up (see Table A1a,b in Appendix A).

## 4. Discussion

This study investigated whether baseline demographic, clinical, and psychosocial characteristics could predict treatment outcomes or drop-out in a cohort of female adolescents under 16 years of age with anorexia nervosa (AN) who did undertake an intensive 20-week course of enhanced cognitive behavioral therapy (CBT-E). Four main findings emerged.

First, among patients who completed inpatient treatment and the 20-week follow-up assessment, a higher baseline BMI-for-age percentile was associated with an increased likelihood of achieving a “good BMI outcome” at the end of inpatient treatment (EOIT). This finding mirrors those observed in adult patients with AN undergoing CBT-E, reinforcing the importance of initiating inpatient CBT-E at an earlier stage—when body weight is not yet critically low—in young adolescent patients [28]. In addition, we found that lower baseline clinical impairment predicted both a higher BMI-for-age percentile and lower eating disorder psychopathology at EOIT, while higher baseline general psychopathology predicted better treatment outcomes at follow-up. These findings deserve attention and, if confirmed, would support the need for early interventions that prevent severely compromising situations that are difficult to reverse. Finally, greater weight regain from baseline to EOIT was associated with a higher BMI-for-age percentile at follow-up, underscoring the importance of prioritizing weight restoration during inpatient treatment.

Second, in the intention-to-treat analysis, no baseline demographic or clinical characteristics were associated with treatment outcome at EOIT or 20-week follow-up, in line with prior studies evaluating CBT-E in adolescents [26,46]. The sole exception was age: younger adolescents (particularly those aged 11–13) exhibited greater levels of eating disorder psychopathology at follow-up, although their weight outcomes were comparable to older participants. This finding, not previously reported, raises the possibility that early adolescent patients may benefit from tailored enhancements to standard CBT-E—especially with regard to addressing the overvaluation of weight and shape, body image concerns, and dietary restraint. Additional strategies may also be needed during the final treatment phase to support transition and prevent relapse.

The discrepancy between intention-to-treat and per-protocol analysis is challenging to explain. However, when predictor variables show different relationships with the outcome in these two approaches, it highlights the role of adherence. If a predictor has a more substantial impact on the outcome in the per-protocol analysis (where adherence is high), it could suggest that the treatment’s effectiveness is dependent on the patient following the protocol [47].

Third, no baseline variables were found to predict treatment drop-out. This finding matches the literature about CBT-E in both adolescent and adult populations [46]. While previous meta-analyses identified motivation to change as a significant predictor of attrition [48], motivation was not formally assessed in the present study. Nevertheless, our treatment model includes a structured pre-admission process comprising motivational enhancement and informed commitment to weight restoration. This may have contributed to the low drop-out rate (16.2%) and may help explain the absence of predictors.

Fourth, treatment outcomes were positive and sustained. Among those who completed treatment, substantial improvements were observed in weight restoration, specific eating disorder psychopathology, general distress, and functional impairment. These gains were widely held at follow-up, even in the absence of extensive additional care. Mixed-effects models confirmed a stable trajectory of improvement across time points. Over 90% of patients accomplished a “good BMI outcome” and more than 70% met full response criteria at the end of treatment; at follow-up, these figures remained relatively high, at 64.7% and 55.9%, respectively. These promising outcomes, although requiring confirmation through a randomized controlled trial, are comparable to those reported in an earlier study involving older adolescents [49].

As far as we know, this is the first and only study to evaluate outcomes of intensive CBT-E in a sample consisting exclusively of younger adolescent females with AN. Despite its strengths—including a real-world clinical setting, a manualized treatment approach, and validated outcome measures—some limitations warrant consideration. First, the modest sample size limited statistical power to identify subtle predictor effects. Second, the relatively short follow-up period precludes conclusions about long-term outcomes and durability of treatment effects. Third, relevant psychological predictors such as co-morbid psychopathology, motivation to change, personality traits, and the quality of the parental support or the cohesion of family relationships were not assessed. Finally, the influence of post-discharge outpatient CBT-E sessions on follow-up outcomes could not be evaluated due to resource constraints.

## 5. Conclusions

Intensive CBT-E appears to be a highly effective treatment for younger adolescents with AN, producing robust improvements in body weight and psychopathology that are largely sustained over time. Some clinical variables seem to influence treatment outcomes in patients who completed both inpatient and follow-up assessments, such as baseline body weight, clinical impairment, general psychopathology, and weight regain. Moreover, in the overall sample, younger age may be associated with greater residual psychopathology at follow-up. These findings underscore the need for an early intervention, a specific focus on weight regain, and a potential adaptation to the standard CBT-E model in early adolescence. Further research using larger samples and extended follow-up is needed to replicate and expand upon these findings, especially given the clinical importance of optimizing treatment for this vulnerable age group.

## Figures and Tables

**Table 1:** Demographic and baseline characteristics of 68 adolescent patients with anorexia nervosa.

Gender, Female, N (%)	100%
Age, years, mean (SD)	14.6 (1.0)
BMI-for-age percentile, mean (SD)	5.0 (7.6)
Age onset, years, mean (SD)	12.9 (1.6)
Illness duration, years, mean (SD)	1.8 (1.3)
Educational level, N (%)	
Elementary school	20 (29.4)
Middle school certificate	48 (70.6)

**Table 2:** Mean (SE) of baseline, end of intensive treatment—inpatient followed by day patient—(EOIT) and the 20-week follow-up (20-week FU) data in 68 adolescent patients (≤16 years) with anorexia nervosa. An intent-to-treat analysis with a multiple imputation procedure was used. Pooled data are presented.

	Mean and (SE)			Analysis of Variance for Repeated Measures	Linear Mixed Model	
	Baseline ^a^	EOIT ^b^	20-Week FU ^c^	Pairwise Comparisons *	Linear Growth	Quadratic Growth
BMI-for-age percentile	5.0 (0.9)	49.2 (2.3)	41.2 (7.9)	a < b,c; b > c	β = 167.98 *t* = 7.90, *p* < 0.001	β = −149.62, *t* = 6.43, *p* < 0.001
Expected Body Weight	77.1 (1.2)	101.8 (1.9)	99.1 (3.7)	a < b,c; b > c	β = 92.19 *t* = 20.92, *p* < 0.001	β =−73.33, *t* = 17.18, *p* < 0.001
Eating Disorder Examination Questionnaire						
Restraint	4.1 (0.2)	0.9 (0.3)	1.4 (0.5)	a > b,c; b < c	β = −12.40, *t* = 6.48, *p* < 0.001	β = 10.96, *t* = 4.72, *p* < 0.001
Eating concern	3.4 (0.1)	1.3 (0.1)	1.2 (0.2)	a > b,c	β = −7.69, *t* = 9.95, *p* < 0.001	β = 6.15, *t* = 5.96, *p* < 0.001
Weight concern	4.0 (0.2)	2.1 (0.2)	1.8 (0.2)	a > b,c; b > c	β = −6.83, *t* = 8.30, *p* < 0.001	β = 5.02, *t* = 5.05, *p* < 0.001
Shape concern	4.8 (0.1)	3.3 (0.2)	2.9 (0.2)	a > b,c; b > c	β = −4.48, *t* = 4.94, *p* < 0.001	β = 2.67, *t* = 2.46, *p* = 0.014
Global score	4.1 (0.1)	2.1 (0.2)	1.9 (0.2)	a > b,c	β = −7.85, *t* = 10.52, *p* < 0.001	β = 6.20, *t* = 7.22, *p* < 0.001
Brief Symptom Inventory						
Global score	2.1 (0.1)	1.2 (0.2)	1.1 (0.5)	a > b,c	β = −3.10, *t* = 2.85, *p* = 0.006	β = 2.31, *t* = 1.57, *p* = 0.140
Clinical Impairment Assessment						
Global score	33.7 (1.3)	16.4 (1.8)	15.2 (2.7)	a > b,c	β = −60.27, *t* = 6.83, *p* < 0.001	β = 45.68, *t* = 4.03, *p* < 0.001

* Indicate significant differences (*p* < 0.05) between baseline (a), EOIT (b) and 20-week FU (c).

**Table 3:** (**a**) Multivariable logistic regression analysis of the 20-week follow-up (20-week FU) in adolescent patients (≤16 years) with anorexia nervosa treated via intensive enhanced cognitive behavior therapy (CBT-E). Completers’ analysis, including the 40 patients who completed the 20-week FU assessment. (**b**) Multivariate linear regression analysis of body mass index (BMI) and eating disorder psychopathology at the end of intensive treatment—inpatient followed by day patient—(EOIT) and the 20-week follow-up (20-week FU) in adolescent patients (≤16 years) with anorexia nervosa treated via intensive enhanced cognitive behavior therapy (CBT-E). Completers’ analysis, including the 40 patients who completed the 20-week FU assessment.

(a)				
	Dependent Variable: Good BMI Outcome at 20-Week FU			
Independent variables	*β*	*p*-Value	OR	95% CI OR
Age	−0.63	0.379	0.53	0.13–2.17
Duration of illness	−0.001	0.999	0.99	0.34–2.99
BMI-for-age percentile	1.24	0.035	3.44	1.09–10.88
EDE-Q—global score	−1.91	0.309	0.15	0.004–5.88
BSI—global score	2.60	0.269	13.50	0.13–1359.14
CIA—global score	−0.08	0.661	0.93	0.66–1.30
Delta BMI-for-age percentile	−0.06	0.188	0.94	0.86–1.03
Delta EDE-Q	0.68	0.504	1.97	0.27–14.44
Delta BSI	−2.73	0.174	0.06	0.001–3.34
Delta CIA	0.19	0.203	1.21	0.90–1.61
	**Dependent variable: Full response at 20-week FU**			
Independent variables	*β*	*p*-value	OR	95% CI OR
Age	−0.08	0.882	0.92	0.31–2.76
Duration of illness	−0.43	0.415	0.65	0.23–1.83
BMI-for-age percentile	0.55	0.083	1.74	0.93–3.24
EDE-Q—global score	−3.03	0.116	0.05	0.001–2.12
BSI—global score	3.90	0.053	49.26	0.95–2544.12
CIA—global score	−0.07	0.656	0.93	0.68–1.27
Delta BMI-for-age percentile	−0.03	0.350	0.97	0.90–1.04
Delta EDE—Q	1.21	0.277	3.37	0.38–29.98
Delta BSI	−2.75	0.075	0.06	0.003–1.32
Delta CIA	0.23	0.094	1.26	0.96–1.66
**(b)**				
	**Dependent variable: BMI-for-age percentile at EOIT**			
Independent variables	*β*	*t*	*p*-value	95% CI
Age	−0.27	1.68	0.103	−9.29–0.90
Duration of illness	−0.03	0.19	0.852	−3.98–3.30
BMI-for-age percentile	−0.16	0.95	0.349	−1.63–0.59
Eating Disorder Examination Questionnaire (EDE-Q)—global score	0.31	1.08	0.289	−3.94–12.81
Brief symptom Inventory (BSI)—global score	0.24	0.97	0.341	−6.08–17.06
Clinical Impairment Assessment (CIA)—global score	−0.72	2.53	0.017	−2.08–−0.22
	**Dependent variable: EDE-Q global score at EOIT**			
Independent variables	*β*	*t*	*p*-value	95% CI
Age	−0.11	0.77	0.447	−0.41–0.18
Duration of illness	−0.16	1.58	0.125	−0.38–0.05
BMI-for-age percentile	0.09	0.56	0.578	−0.05–0.08
Eating Disorder Examination Questionnaire (EDE-Q)—global score	−0.46	1.65	0.108	−0.89–0.09
Brief symptom Inventory (BSI)—global score	0.44	1.91	0.066	−0.04–1.31
Clinical Impairment Assessment (CIA)—global score	0.66	2.54	0.016	0.01–0.12
	**Dependent variable: BMI-for-age percentile at 20-week FU**			
Independent variables	*β*	*t*	*p*-value	95% CI
Age	−0.09	0.60	0.550	−9.96–5.42
Duration of illness	−0.09	0.60	0.552	−7.07–3.86
BMI-for-age percentile	0.21	1.21	0.237	−0.73–2.84
EDE-Q—global score	−0.53	0.98	0.337	−34.52–12.22
BSI—global score	0.56	2.15	0.040	0.94–39.09
CIA—global score	−0.12	0.27	0.786	−2.47–1.89
Delta BMI-for-age percentile	−0.46	2.68	0.012	−1.22–−0.16
Delta EDE-Q	0.47	1.17	0.250	−7.25–26.75
Delta BSI	−0.31	1.66	0.108	−23.61–2.45
Delta CIA	0.06	0.18	0.857	−1.60–1.91
	**Dependent variable: EDE-Q global score at 20-week FU**			
Independent variables	*β*	*t*	*p*-value	95% CI
Age	−0.18	1.25	0.222	−0.68–0.16
Duration of illness	0.23	1.59	0.122	−0.07–0.53
BMI-for-age percentile	−0.04	0.26	0.799	−0.11–0.09
EDE-Q—global score	0.51	0.99	0.330	−0.66–1.90
BSI—global score	−0.56	2.26	0.032	−2.20–−0.11
CIA—global score	0.74	1.85	0.075	−0.01–0.23
Delta BMI-for-age percentile	−0.11	0.68	0.501	−0.04–0.02
Delta EDE-Q	−0.48	1.24	0.224	−1.50–0.37
Delta BSI	0.32	1.81	0.081	−0.08–1.35
Delta CIA	−0.51	1.68	0.107	−0.17–0.02

Delta indicates the difference between baseline and end of treatment (EOT). Delta indicates the difference between baseline and EOIT.

## Data Availability

The data supporting this study’s findings are available from the corresponding author, S.C., upon reasonable request.

## Appendix A

**Table A1:** (**a**) Multivariable logistic regression analysis of drop-out and the end of intensive treatment—inpatient followed by day patient—(EOIT) and the 20-week follow-up (20-week FU) in 68 adolescent patients (≤16 years) with anorexia nervosa treated via intensive enhanced cognitive behavior therapy (CBT-E). Intention-to-treat analysis with multiple imputation procedure was used. Pooled data are presented. (**b**) Multivariate linear regression analysis of body mass index (BMI) and eating disorder psychopathology at the end of intensive treatment—inpatient followed by day patient” (EOIT) and the 20-week follow-up (20-week FU) in 68 adolescent patients (≤16 years) with anorexia nervosa treated via intensive enhanced cognitive behavior therapy (CBT-E). Intention-to-treat analysis with multiple imputation procedure was used. Pooled data are presented.

(a)				
	Dependent Variable: Drop-Out Rate			
Independent variables	*β*	*p*-value	OR	95% CI OR
Age	0.13	0.766	1.14	0.49–2.65
Duration of illness	0.01	0.984	1.01	0.57–1.78
BMI-for-age percentile	−0.05	0.388	0.95	0.83–1.07
Eating Disorder Examination Questionnaire (EDE–Q)—global score	1.34	0.086	3.81	0.83–17.54
Brief symptom Inventory (BSI)—global score	−0.48	0.578	0.62	0.11–3.41
Clinical Impairment Assessment (CIA)—global score	−0.06	0.411	0.94	0.81–1.09
	**Dependent variable: Good BMI outcome at EOIT**			
Independent variables	*β*	*p*-value	OR	95% CI OR
Age	0.22	0.808	1.25	0.20–7.65
Duration of illness	−0.45	0.270	0.64	0.29–1.42
BMI-for-age percentile	12.1	0.988	0.00	0.00
EDE–Q—global score	−0.25	0.834	0.77	0.07–8.92
BSI—global score	0.23	0.839	1.26	0.13–12.20
CIA—global score	−0.06	0.664	0.94	0.69–1.27
	**Dependent variable: Full response at EOIT**			
Independent variables	*β*	*p*-value	OR	95% CI OR
Age	0.22	0.562	1.25	0.59–2.67
Duration of illness	−0.06	0.813	0.94	0.59–1.52
BMI-for-age percentile	0.03	0.504	1.03	0.94–1.14
EDE–Q—global score	−0.22	0.691	0.80	0.27–2.39
BSI—global score	0.47	0.470	1.60	0.45–5.72
CIA—global score	−0.08	0.244	0.92	0.80–1.06
	**Dependent variable: Good BMI outcome at 20-week FU**			
Independent variables	*β*	*p*-value	OR	95% CI OR
Age	−0.03	0.951	0.97	0.33–2.85
Duration of illness	0.04	0.903	1.04	0.56–1.93
BMI-for-age percentile	0.13	0.394	1.14	0.82–1.60
EDE–Q—global score	−0.18	0.890	0.83	0.05–14.46
BSI—global score	1.45	0.270	4.28	0.29–63.14
CIA—global score	−0.11	0.311	0.89	0.71–1.12
Delta BMI-for-age percentile	−0.06	0.165	0.94	0.87–1.03
Delta EDE-Q	0.36	0.664	1.44	0.26–7.95
Delta BSI	−1.25	0.332	0.29	0.02–4.15
Delta CIA	0.08	0.415	1.08	0.89–1.31
	**Dependent variable: Full response at 20-week FU**			
Independent variables	*β*	*p*-value	OR	95% CI OR
Age	0.36	0.391	1.44	0.62–3.33
Duration of illness	−0.12	0.689	0.89	0.49–1.61
BMI-for-age percentile	0.11	0.409	1.11	0.85–1.46
EDE-Q—global score	−0.46	0.681	0.63	0.06–6.46
BSI—global score	1.98	0.178	7.30	0.36–148.01
CIA—global score	−0.15	0.081	0.86	0.72–1.02
Delta BMI-for-age percentile	−0.03	0.338	0.96	0.89–1.04
Delta EDE–Q	0.34	0.722	1.40	0.19–10.13
Delta BSI	−1.79	0.093	0.17	0.02–1.37
Delta CIA	0.14	0.155	1.15	0.94–1.40
**(b)**				
	**Dependent variable: BMI-for-age percentile at EOIT**			
Independent variables	*β*	*t*	*p*-value	95% CI
Age	−4.12	1.60	0.110	−9.17–0.94
Duration of illness	−1.27	0.67	0.501	−4.99–2.45
BMI-for-age percentile	−0.18	0.47	0.639	−0.94–0.58
Eating Disorder Examination Questionnaire (EDE-Q)—global score	3.12	0.81	0.425	−4.72–10.96
Brief symptom Inventory (BSI)—global score	4.47	1.01	0.312	−4.22–13.17
Clinical Impairment Assessment (CIA)—global score	−0.79	1.78	0.082	−1.68–0.10
	**Dependent variable: EDE-Q global score at EOIT**			
Independent variables	*β*	*t*	*p*-value	95% CI
Age	0.02	0.10	0.920	−0.31–0.34
Duration of illness	0.02	0.18	0.858	−0.21–0.25
BMI-for-age percentile	−0.02	1.07	0.287	−0.07–0.02
Eating Disorder Examination Questionnaire (EDE-Q)—global score	0.19	0.85	0.398	−0.25–0.63
Brief symptom Inventory (BSI)—global score	−0.01	0.03	0.973	−0.55–0.53
Clinical Impairment Assessment (CIA)—global score	0.02	0.58	0.561	−0.04–0.07
	**Dependent variable: BMI-for-age percentile at 20-week FU**			
Independent variables	*β*	*t*	*p*-value	95% CI
Age	0.93	0.10	0.917	−17.58–19.43
Duration of illness	−0.89	0.15	0.880	−13.13–11.34
BMI-for-age percentile	1.44	0.91	0.373	−1.99–4.88
EDE-Q—global score	−5.57	0.20	0.849	−72.46–61.32
BSI—global score	21.85	1.25	0.231	−15.73–59.44
CIA—global score	−0.99	0.48	0.640	−5.53–3.55
Delta BMI-for-age percentile	−0.72	1.14	0.283	−2.13–0.70
Delta EDE-Q	7.37	0.38	0.716	−37.87–52.60
Delta BSI	−18.92	0.97	0.360	−64.11–26.27
Delta CIA	0.57	0.32	0.754	−3.41–4.55
	**Dependent variable: EDE-Q global score at 20-week FU**			
Independent variables	*β*	*t*	*p*-value	95% CI
**Age**	**−0.38**	**2.07**	**0.039**	**−0.75–−0.02**
Duration of illness	0.12	0.80	0.427	−0.17–0.41
BMI-for-age percentile	−0.02	0.71	0.482	−0.09–0.04
EDE-Q—global score	0.10	0.26	0.793	−0.65–0.84
BSI—global score	−0.53	1.41	0.164	−1.27–0.22
CIA—global score	0.09	2.03	0.052	−0.001–0.18
Delta BMI-for-age percentile	0.004	0.35	0.726	−0.02–0.03
Delta EDE-Q	−0.19	0.65	0.519	−0.80–0.41
Delta BSI	0.57	1.66	0.109	−0.14–1.27
Delta CIA	−0.05	1.30	0.212	−0.14–0.03

Delta indicates the difference between baseline and end of treatment (EOT). Delta indicates the difference between baseline and EOIT.
